# A nanoemulsion-adjuvanted trivalent neuraminidase vaccine confers cross-protection against divergent influenza viruses

**DOI:** 10.3389/fcimb.2026.1931757

**Published:** 2026-09-14

**Authors:** Jiaxin Bai, Shuang Li, Chen Zhao, Ting Yin, Diansong Li, Lirui Han, Yongbo Qiao, Yehong Wu

**Affiliations:** 1 Changchun Institute of Biological Products Co., Ltd, Changchun, China; 2 State Key Laboratory of Novel Vaccines for Emerging Infectious Diseases, China National Biotec Group Company Limited, Beijing, China

**Keywords:** baculovirus, NA-inhibiting antibody, nanoemulsion adjuvant, neuraminidase, universal influenza vaccines

## Abstract

Developing universal influenza vaccines (UIVs) capable of eliciting broad protection remains a critical public health priority. Neuraminidase (NA), a relatively conserved surface antigen of influenza viruses, is a promising target for UIV design. Here, we produced full-length trivalent NA proteins via a baculovirus-insect cell expression system, matching the WHO-recommended strains (H1N1, H3N2, and B/Victoria) for the 2024–2025 season. Immunogenicity evaluation of N2NA (derived from H3N2) in mice demonstrated that nanoemulsion (NE) adjuvant significantly enhanced NA-inhibiting (NI) antibody titers, CD4 T cell responses, and the numbers of IL-2-, IL-4- and IFN-γ-secreting lymphocytes compared to the unadjuvanted vaccine. Monovalent N1 NA and BV NA with NE adjuvant similarly induced stronger NI antibody responses and antiviral protection. Furthermore, the NE-adjuvanted trivalent NA vaccine (N1+N2+BV) elicited robust humoral and cellular immunity and provided effective protection in mice against two H1N1, two B/Victoria, and one H5N1 strain. Collectively, these findings support the efficacy of a multivalent NA-based vaccine strategy and highlight its strong potential as a candidate UIV.

## 1 Introduction

Influenza viruses cause high annual morbidity and mortality worldwide, imposing a substantial economic and societal burden. Beyond seasonal epidemics, pandemics pose an even greater threat. Currently, the highly pathogenic avian influenza H5N1 virus circulates in wild birds and mammals, with sporadic spillover infections reported in humans, raising concerns about the next pandemic (Cargnin Faccin and Perez, 2024). As enveloped, segmented negative-sense RNA viruses with 8 genome segments, influenza viruses have high genetic plasticity, enabling frequent antigenic drift and shift. The viral envelope harbors two major glycoproteins: hemagglutinin (HA) and neuraminidase (NA) (Gamblin and Skehel, 2010; Krammer et al., 2018a). HA mediates viral binding to host cell sialic acid receptors and subsequent membrane fusion, serving as the primary target for eliciting neutralizing antibodies. NA is a type II transmembrane glycoprotein that cleaves sialic acid residues, thereby facilitating progeny virus release, preventing viral aggregation, and promoting viral spread within the host.

Currently licensed influenza vaccines primarily employ HA as the major antigenic component, conferring protection against circulating strains by inducing hemagglutination-inhibiting antibodies. However, the highly variable globular head of HA necessitates annual vaccine strain updates, greatly limiting long-term efficacy. Although conventional vaccines contain some NA antigen, their standardization and evaluation focus on HA, leading to limited and poorly characterized anti-NA immune responses (Wohlbold and Krammer, 2014; Eichelberger et al., 2018). Major reasons include low NA abundance on the viral surface, its weaker immunodominance relative to HA, and lack of standardized immunogenicity assays. Nevertheless, accumulating evidence indicates that anti-NA antibodies can neutralize viruses and correlate with protection in humans (Strohmeier et al., 2022a; Lv et al., 2025). A cohort study of an H3N2 outbreak in unvaccinated military recruits found that neuraminidase inhibition (NI) antibody titers were independently associated with reduced infection risk and exhibited broader reactivity against drifted strains than pseudovirus-neutralizing antibodies (Chen et al., 2018).

Developing universal influenza vaccines (UIVs) that elicit broad and durable protection is pivotal to overcoming the limitations of seasonal vaccines. Most UIV studies concentrate on conserved epitopes in the HA stalk, whereas NA has received relatively little attention as a vaccine antigen. Notably, NA evolves at a far slower rate than the HA head domain, rendering its enzymatic active site highly conserved across influenza subtypes. Previous studies have demonstrated that pre-existing immunity to the 2009 pandemic H1N1 virus, which elicits potent N1 neuraminidase-inhibiting antibodies, confers full protection against clade 2.3.4.4b H5N1 in ferrets (Hansen et al., 2023). Moreover, population serological analyses indicate that human humoral immunity against H5N1 is predominantly mediated by NA-specific antibodies. Additionally, seasonal H1N1 infection markedly enhances cross-reactive antibody responses to bovine-origin H5N1 NA in humans, supporting NA-targeted strategies for the simultaneous control of seasonal and potentially pandemic influenza strains (Sandbulte et al., 2007; Memoli et al., 2016).

In novel vaccine platforms such as recombinant protein subunit vaccines, antigens often require adjuvants to facilitate efficient antigen presentation and elicit potent immune responses. Adjuvants serve to amplify overall immune responsiveness and improve the durability of vaccine-induced protection (Coffman et al., 2010). As an oil-in-water adjuvant, nanoemulsion (NE) has demonstrated favorable safety profiles and strong immune-enhancing effects in influenza vaccines.

Based on the above background, the present study successfully produced full-length recombinant NA proteins corresponding to the three embryonated chicken egg-derived candidate vaccine strains (H1N1, H3N2, and B/Victoria) recommended by the WHO for the 2024–2025 influenza season using the baculovirus-insect cell expression system (BEVS). We systematically evaluated the immunogenicity and protective efficacy of N2 NA, N1 NA, B/Victoria NA, and a trivalent NA combination (N1+N2+BV) in a mouse model, and simultaneously investigated the immune-enhancing effect of nanoemulsion adjuvant on NA-based vaccines. This study aims to provide experimental evidence supporting the development of a multivalent neuraminidase-based combined immunization strategy for UIVs.

## 2 Materials and methods

### 2.1 Cells, mice, viruses

Sf-RVN insect cells (Merck, USA) were propagated in Vigor-S101 serum-free medium (Bioengine, China).

Female 6-week-old BALB/c mice were maintained in a specific pathogen-free environment.

A panel of influenza viruses encompassing diverse subtypes, lineages, and years of isolation (obtained from the National Institute for Biological Standards and Control, UK) was employed for challenge studies. The virus strains and their amino acid similarity to the vaccine antigens are summarized in **Table 1**.

**Table 1:** Influenza virus strains used for challenge protection studies.

Subtype/lineage	Strain designation	Amino acid similarity (%)
H1N1	A/Victoria/4897/2022 A/Guangdong-Maonan/SWL1536/2019	100 96.28
BV	B/Austria/1359417/2021 B/Washington/02/2019	100 98.71
H5N1	A/Vietnam/1194/2004	79.95

Full-length sequences were obtained from the GISAID database and analyzed using Clustal Omega.

### 2.2 Preparation of the recombinant proteins

Recombinant NA proteins were produced using a BEVS (Loganathan et al., 2024). Viral RNA was extracted from A/Victoria/4897/2022 (H1N1), A/Thailand/8/2022 (H3N2), and B/Austria/1359417/2021 (BV) viruses using the Simply P Total RNA Extraction Kit (BioFlux, China). First-strand cDNA synthesis was performed with EasyScript First-Strand cDNA Synthesis SuperMix (TransGen Biotech, China), followed by PCR amplification of NA genes using strain-specific primers (**Table 2**) and TransStart FastPfuFly DNA Polymerase (TransGen Biotech).

**Table 2:** Primers used for amplification of influenza NA genes.

Target	Direction	Sequence (5′→3′)
H1N1 NA	Forward Reverse	tcccaccatcgggcgcGGATCCatgaatccaa accaaaagataataaccattgg gcttggtaccgcatgcCTCGAGttaattgtca atggtgaatggcaactcag
H3N2 NA	Forward Reverse	tcccaccatcgggcgcGGATCCatgaatccaa atcaaaagataataacgattggc agcttggtaccgcatgcCTCGAGttatgtatgc atgagactgaggttcgc
BV NA	Forward Reverse	tcccaccatcgggcgcGGATCCatgctacctt caactatacaaacgttaacc ttggtaccgcatgcCTCGAGttacagagcca tatcaacacctgtgatag

Uppercase letters indicate restriction enzyme sites (*BamHI/XhoI*).

Amplified NA fragments were cloned into the pFastBac1 vector (Thermo Fisher Scientific, USA) using the Seamless Cloning Kit (Beyotime, China). Positive clones were selected in LB medium, and plasmid integrity was verified by sequencing (Comate Bioscience, China). Verified constructs were transformed into DH10Bac™ E. coli competent cells (Invitrogen, USA) to generate recombinant bacmids.

Recombinant bacmids were transfected into Sf-RVN cells using BaculoFectin™ II transfection reagent (Oxford Expression Technologies, UK). After 5 days, P1 viral stocks were harvested and amplified in Sf-RVN cells cultured in Vigor-S101 serum-free medium to generate high-titer P2 virus stocks. For protein expression, Sf-RVN cells were infected at an MOI of 1.0 and incubated at 27°C for 72 h. Cell pellets were resuspended in PBS containing 1% Triton X-100 (Sigma-Aldrich, USA) and incubated for 1 hr at 4°C with gentle agitation.

The lysate was clarified by centrifugation, then sequentially purified using Q Sepharose FF chromatography (Cytiva, USA) and Lentil Lectin affinity chromatography (Cytiva, USA). Purified proteins were buffer-exchanged into PBS using 100-kDa centrifugal concentrators (Merck Millipore, UK). Protein concentration was determined by BCA assay (Beyotime, China), and aliquots were stored at -80°C.

### 2.3 Characterization of NA

Purified recombinant NA proteins (N1NA, N2NA, BVNA) were systematically characterized in terms of purity, oligomeric state, structural integrity, and functional activity. Protein purity was evaluated by reducing SDS-PAGE, with bands visualized using Coomassie brilliant blue R-250 staining (Beyotime, China). To identify glycosylation modifications, recombinant NA proteins were deglycosylated by incubation with peptide N-glycosidase F (PNGase F; NEB, USA) at 37°C for 1 h. The digested products were then denatured and analyzed by reducing SDS-PAGE. The oligomeric state of the recombinant proteins was subsequently assessed by non-reducing SDS-PAGE.

Neuraminidase enzymatic activity was quantified using a fetuin-based ELISA. Briefly, 96-well plates were coated with 100 µL/well of fetuin (25 µg/mL; MedChemExpress, USA) in 0.05 M carbonate buffer and incubated at 4°C for 18 h. After blocking, and washing with PBST (0.05% Tween-20), 100 µL of NA protein (1 µg/well) or PBS control was added and incubated 16–18 h at 37°C. Following PBST washes, 100 µL HRP-conjugated peanut agglutinin (PNA, 5 µg/mL; Sigma-Aldrich) was added for 2 h at 37°C. After five PBST washes, 100 µL TMB substrate (Tiangen Biotech, China) was added for 10 min at room temperature, and reactions were terminated with 100 µL 1M HCl. Absorbance at 450 nm was measured using a SpectraMax iD3 reader.

### 2.4 Immunization

All animal procedures complied with China’s National Animal Management Regulations and were approved by the Institutional Animal Care and Use Committee of Changchun Institute of Biological Products (Permit No: CCIBP202511-05). The NE adjuvant was provided by Changchun Institute of Biological Products. All immunizations were administered via intramuscular injection in a 100 µL volume per hind limb.

#### 2.4.1 Adjuvant and regimen screening

To evaluate the immunization regimens and adjuvant efficacy, BALB/c mice (n = 6 per group) were divided into five groups as detailed in **Table 3**. Mice were administered either a single immunization or prime-boost immunization (day 0 + day 21) with vaccines containing 5 µg of N2NA, with or without nanoemulsion adjuvant. Serum samples were collected before immunization (day 0), after priming (day 21) and post-boost (day 35) to determine NA-specific binding antibody levels and NI antibody titers. Furthermore, mice were euthanized on day 35, splenic lymphocytes were isolated, flow cytometry was used to detect the levels of CD4^+^ and CD8^+^ T cells, and ELISpot assays were performed to quantify the numbers of IL-2-, IL-4-, and IFN-γ-secreting lymphocytes.

**Table 3:** Adjuvant and regimen screening groups.

Groups	Prime (day 0)	Boost (day 21)
1	5 µg N2NA	–
2	5 µg N2NA + NE	–
3	5 µg N2NA	5 µg N2NA
4	5 µg N2NA + NE	5 µg N2NA + NE
5	PBS	PBS

#### 2.4.2 Dose-response evaluation

Based on the optimal immunization regimen identified in Section 2.4.1, a prime-boost schedule (day 0 + day 21) was used in this part. Three N2 NA doses (0.2 µg, 1 µg, and 5 µg) with or without NE adjuvant were administered to explore the dose-dependent immunogenicity of the vaccine, as detailed in **Table 4**. On day 35, serum samples were collected to measure NA-specific binding and NI antibody titers, and splenic lymphocytes were isolated for the analysis of CD4^+^/CD8^+^ T cell subsets, to systematically characterize the humoral and cellular immune responses at different antigen doses.

**Table 4:** Dose-response groups.

Groups	Prime (day 0)	Boost (day 21)
6	0.2 µg N2NA	0.2 µg N2NA
7	0.2 µg N2NA + NE	0.2 µg N2NA + NE
8	1 µg N2NA	1 µg N2NA
9	1 µg N2NA + NE	1 µg N2NA + NE
3*	5 µg N2NA	5 µg N2NA
4*	5 µg N2NA + NE	5 µg N2NA + NE

*Groups 3 & 4 from **Table 3** included for dose comparison.

#### 2.4.3 Monovalent N1NA/BVNA immunogenicity

N1NA and BVNA immunogenicity was evaluated using prime-boost regimens (5 µg antigen ± NE; Days 0/21). Serum was collected on Day 35 to assess NA-specific antibody and NI antibody titers. For protective efficacy evaluation, mice were intranasally challenged on day 42 as detailed in **Table 5**: the N1NA-immunized groups were challenged with 10 × LD_50_ of the homologous strain A/Victoria/4897/2022 (H1N1) and the heterosubtypic strain A/Vietnam/1194/2004 (H5N1), respectively, whereas the BV NA-immunized groups were challenged with 10 × LD_50_ of the homologous strain B/Austria/1359417/2021.

**Table 5:** Monovalent N1NA/BVNA experimental design.

Groups	Prime (day 0)	Boost (day 21)	Challenge (day 42)
10	5 µg N1NA	5 µg N1NA	A/Victoria/4897/2022 (H1N1) A/Vietnam/1194/2004 (H5N1)
11	5 µg N1NA + NE	5 µg N1NA + NE	A/Victoria/4897/2022 (H1N1) A/Vietnam/1194/2004 (H5N1)
12	5 µg BVNA	5 µg BVNA	B/Austria/1359417/2021 (BV)
13	5 µg BVNA+ NE	5 µg BVNA+ NE	B/Austria/1359417/2021 (BV)
14	PBS	PBS	A/Victoria/4897/2022 (H1N1)
15	PBS	PBS	A/Vietnam/1194/2004 (H5N1)
16	PBS	PBS	B/Austria/1359417/2021 (BV)

#### 2.4.4 Trivalent vaccine evaluation

To evaluate the cross-protective immune efficacy of the trivalent NA vaccine, mice were immunized using a prime-boost regimen (Days 0/21) with the trivalent neuraminidase vaccine containing 5 μg of N1NA, N2NA, and BVNA each, divided into NE-adjuvanted and non-adjuvanted groups as detailed in **Table 6**. On day 35, serum samples were collected to determine NA-specific antibody levels and NI antibody titers, and splenic lymphocytes were isolated for the analysis of CD4^+^/CD8^+^ T cell subsets and IL-2-, IL-4-, and IFN-γ-secreting lymphocytes. For cross-protection evaluation, mice were intranasally challenged on day 42 with 10 × LD_50_ of homologous and heterologous influenza viruses representing three distinct lineages, including H1N1, H5N1, and BV strains.

**Table 6:** Trivalent vaccine experimental design.

Groups	Prime (day 0)	Boost (day 21)	Challenge (day 42)
17	Trivalent NA	Trivalent NA	A/Victoria/4897/2022 (H1N1) A/Guangdong-Maonan/SWL1536/2019 (H1N1) B/Austria/1359417/2021 (BV) B/Washington/02/2019 (BV) A/Vietnam/1194/2004 (H5N1)
18	Trivalent NA + NE	Trivalent NA + NE	
19	PBS	PBS	

Each group of mice was divided into subgroups and challenged with each of the five indicated viral strains, respectively.

### 2.5 Enzyme-linked immunosorbent assay (ELISA)

NA-specific antibody titers in immune sera were quantified by ELISA. Briefly, 96-well plates were coated with 1 μg/mL purified full-length NA (100 μL/well) and incubated overnight at 4°C, then blocked with 5% bovine serum albumin in PBS (BSA-PBS) at 37°C for 2 h. Serially 2-fold diluted sera were added and incubated for 2 h at 37°C. Following PBST washes, HRP-conjugated goat anti-mouse IgG (1:5,000; Dingguo, China) was added per well for 1 h at 37°C. Following the final wash, TMB substrate (Tiangen Biotech, China) was added for 15 min (protected from light). Reactions were stopped with 50 μL 2M H_2_SO_4_, and absorbance at 450 nm was immediately measured using a SpectraMax iD3 plate reader (Molecular Devices, USA). Endpoint titers were defined as the highest serum dilution yielding OD_450_ values ≥ 2-fold above the mean of PBS-immunized control wells. All samples were tested in duplicate.

### 2.6 Neuraminidase inhibition assay

NI antibody titers were determined using a fetuin-based enzymatic assay as previously described with modifications (Strohmeier et al., 2022b). Briefly, 96-well plates were coated with fetuin (25 µg/mL in 0.05 M carbonate buffer, pH 9.6) overnight at 4°C. After blocking with 5% BSA-PBS (2 h, 37°C), serial dilutions of heat-inactivated sera were mixed with a standardized dose of purified full-length NA protein (pre-titrated to optimal working concentration in NA diluent: 0.01 M PBS supplemented with 0.5% BSA), and incubated in fetuin-coated plates for 18 h at 37°C. After washing with PBST, HRP-labeled PNA was added and incubated at 37°C for 2 h. Following the final wash, TMB substrate was added, reactions stopped with 1M HCl, and absorbance measured at 450 nm. The percent inhibition was calculated as:


$$\left[1-(\text{O}{\text{D}}_{450}\text{ sample}/\text{O}{\text{D}}_{450}\text{ virus‐only control})\right]\;\times \;100.$$

NI titers (50% inhibitory concentration, IC_50_) were defined as the reciprocal ofthe highest serum dilution achieving ≥ 50% inhibition. All assays included virus-background and serum-control wells, with validation requiring negative control OD_450_ ≤ 0.1.

### 2.7 Splenocyte isolation and flow cytometry

Splenic lymphocytes from mice were isolated using the Mouse Spleen Lymphocyte Isolation Kit (Solarbio, China) according to the manufacturer’s instructions. Isolated lymphocytes were resuspended in cell staining buffer (Biolegend, USA), followed by staining with fluorochrome-conjugated monoclonal antibodies (Biolegend, USA) for 30 min on ice. After washing with staining buffer, 5 µL 7-aminoactinomycin D was added. Detection was performed using a BD Accuri C6 Plus flow cytometer (BD Biosciences, USA), and data were acquired and analyzed with flow cytometry software to determine the proportion of CD3^+^CD4^+^ and CD3^+^CD8^+^ T cell subsets. The following antibodies were used: anti-CD3 (FITC), anti-CD4 (PE) and anti-CD8 (APC).

### 2.8 Enzyme-linked immunospot (ELISpot) assay

The ELISpot Plus Kit (Mabtech, Sweden) was used to quantitatively detect positive T cells secreting IL-2, IL-4 and IFN-γ in splenic lymphocytes according to the manufacturer’s instructions. For the monovalent N2NA immunogenicity study, purified N2NA protein (10 µg/mL) was used as the stimulant. For the trivalent NA vaccine study, a mixture of N1NA, N2NA, and BVNA proteins (10 µg/mL each) was used as the stimulant. A positive control (10 µg/mL phytohemagglutinin) and a negative control (10 µL serum-free medium) were included. After incubation, antigen-specific spots in each well were counted using an AID iSpot Spectrum ELR08IFL reader (AID Diagnostika GmbH, Germany), and the number of positive T cells secreting the four cytokines was analyzed and calculated with AID ELISpot 7.0 software.

### 2.9 Viral challenge protection evaluation

All animal challenge experiments were conducted in an ABSL-2 enhanced (Animal Biosafety Level 2 enhanced) laboratory. Following intranasal challenge with 10×LD50 of influenza virus under anesthesia with 2–3% isoflurane in oxygen, mice were monitored daily for 14 days for weight changes (expressed as percentage of initial body weight), and survival rate. Animals exhibiting a weight loss of 25% or more were humanely euthanized by gradual-fill CO_2_ inhalation (flow rate: 30-50% chamber volume/min, maintained for ≥1 min after respiratory arrest), followed immediately by cervical dislocation to confirm death.

### 2.10 Statistical analysis

All data are presented as the mean ± SD. ANOVA followed by Tukey’s *post hoc* multiple-comparison test was performed using SPSS PASW Statistics. A *P* value <0.05 was regarded as statistically significant.

## 3 Results

### 3.1 Recombinant NA proteins exhibit enzymatic activity

Reducing SDS-PAGE analysis (**Figure 1A**) revealed distinct single bands at approximately 65 kDa for all three recombinant proteins. Following PNGase F digestion, the molecular weights of the proteins shifted to around 58 kDa (**Figure 1B**), confirming that the higher apparent molecular weight under reducing conditions resulted from extensive N-linked glycosylation. Under non-reducing conditions, all three NA proteins appeared as high-molecular-weight species migrating above 180 kDa (**Figure 1C**), indicative of high-order oligomer formation.

**Figure 1:**
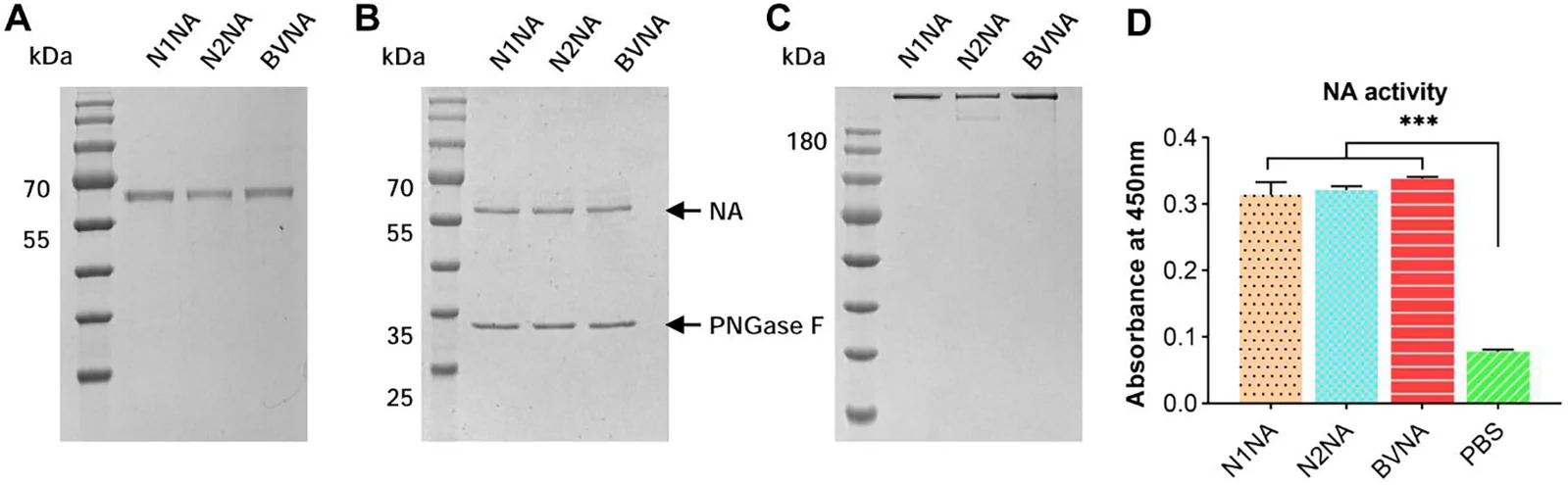
Structural and functional profiling of recombinant NA proteins. **(A)** Reducing SDS-PAGE analysis of purified recombinant NA proteins; **(B)** Reducing SDS-PAGE analysis of recombinant NA proteins after PNGase F digestion; **(C)** Non-reducing SDS-PAGE analysis of recombinant NA proteins; **(D)** Fetuin-based enzymatic activity assay with absorbance detection at 450 nm for NA functional activity. *** indicates P<0.001 (highly significant statistically).

Fetuin-based enzymatic assays confirmed substantial NA activity (**Figure 1D**): recombinant NA efficiently cleaved terminal sialic acid residues from fetuin, enabling exposed galactose residues to bind PNA-HRP and produce detectable colorimetric signals. Collectively, these results verify the native conformation and functional biological activity of the recombinant NA proteins.

### 3.2 NE adjuvanted prime-boost enhances humoral and cellular immunity induced by N2NA vaccine

Using purified N2NA protein consistent with the immunogen as the coating antigen, we systematically evaluated the effects of different immunization regimens and NE adjuvant on the immunogenicity of N2NA vaccine. NA-specific binding antibodies measured by ELISA (**Figure 2A**) revealed that prime-boost (P+B) immunization elicited markedly higher antibody levels than prime-only (P) immunization, and the addition of NE adjuvant further greatly enhanced antibody titers. The N2NA+NE (P+B) group exhibited the highest endpoint antibody titer, approximately 20-fold higher than that in the N2NA (P+B) group. NI antibody titers showed a similar trend (**Figure 2B**), in which both prime-boost strategy and adjuvant incorporation significantly increased functional NA-inhibitory antibody levels, with the highest titer observed in the N2NA+NE (P+B) group, suggesting that this regimen effectively induces NA-specific antibodies with functional inhibitory activity.

**Figure 2:**
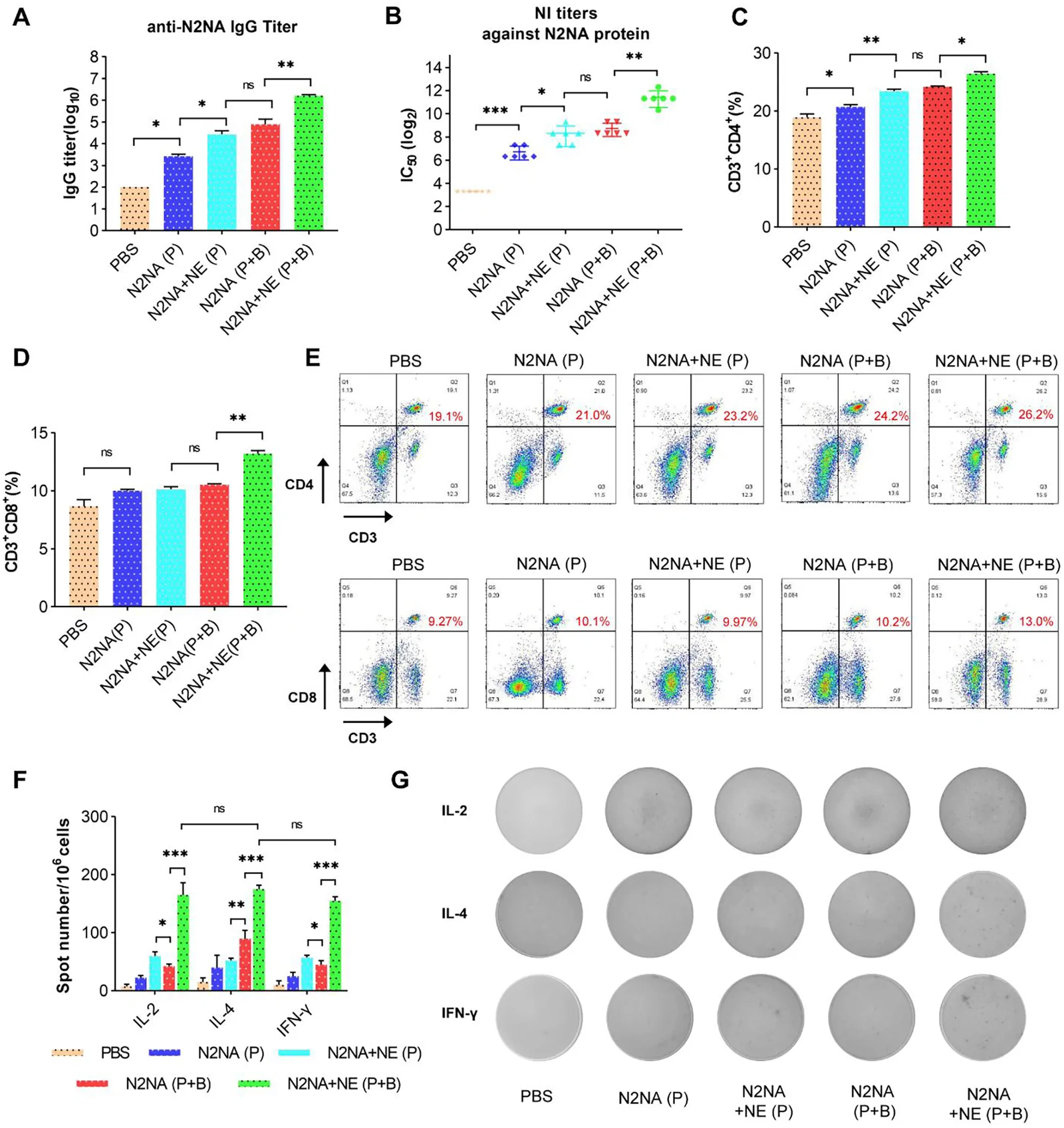
Humoral and cellular immune responses elicited by N2NA vaccine under different immunization regimens with or without NE adjuvant. **(A)** ELISA analysis of NA-specific binding antibody titers; **(B)** NI assay for functional antibody detection; **(C)** Proportions of CD3^+^CD4^+^ T cell subsets; **(D)** Proportions of CD3^+^CD8^+^ T cell subsets; **(E)** Representative flow cytometric plots of T lymphocyte subsets; **(F)** Quantification of cytokine-secreting splenocytes by ELISpot assay; **(G)** Representative ELISpot images of cytokine-secreting lymphocytes. N2NA+NE (P+B), adjuvanted prime-boost; N2NA (P+B), non-adjuvanted prime-boost; N2NA+NE (P), adjuvanted prime-only; N2NA (P), non-adjuvanted prime-only; PBS, blank control; P, prime; B, boost. * indicates P<0.05 (statistically significant). "**" indicates P<0.01 (statistically very significant), "***" indicates P < 0.001 (highly significant statistically), "ns" indicates not significant' (P ≥ 0.05).

Flow cytometric analysis of splenic lymphocyte subsets (**Figures 2C–E**) showed that the percentages of CD3^+^CD4^+^ and CD3^+^CD8^+^ T cells both peaked in the N2NA+NE (P+B) group. No significant difference in CD3^+^CD4^+^ T cells was observed between the N2NA+NE (P) and N2NA (P+B) groups, while CD3^+^CD8^+^ T cells were significantly higher only in the N2NA+NE (P+B) group compared with the PBS control. ELISpot assays using N2NA as the specific stimulant (**Figures 2F, G**) showed that the numbers of IL-2-, IL-4-, and IFN-γ-secreting lymphocytes followed the order of prime-boost > prime-only and adjuvanted groups > non-adjuvanted groups. When comparing the N2NA+NE (P) and N2NA (P+B) groups, the latter displayed higher IL-4 levels but lower IL-2 and IFN-γ levels; moreover, IL-4 expression was markedly higher than IL-2 and IFN-γ in the N2NA (P+B) group, indicating a Th2-biased immune response. The N2NA+NE (P+B) group induced the highest numbers of lymphocytes secreting all three cytokines, with comparable levels of IL-2, IL-4, and IFN-γ, supporting a balanced Th1/Th2 cellular immune response and optimal immunization efficacy.

### 3.3 Dose-dependent enhancement of N2NA vaccine immunogenicity and adjuvant effects of NE on low-dose antigen

To systematically evaluate the effects of antigen dosage and adjuvant on humoral and cellular immune responses, prime-boost immunization was performed using three N2NA doses (0.2 μg, 1 μg, 5 μg) with or without NE adjuvant. ELISA results (**Figure 3A**) showed a significant dose-dependent increase in antibody levels along with rising N2NA dosage, and NE-adjuvanted groups yielded markedly higher titers than non-adjuvanted groups at the same antigen dose. Notably, the 1 μg N2NA+NE group induced higher antibody titers than the 5 μg non-adjuvanted N2NA group, and the 5 μg N2NA+NE group achieved the strongest response. NI antibody titers (**Figure 3B**) showed an identical trend, with the highest level also observed in the 5 μg N2NA+NE group, confirming that the combination of NE and optimal antigen dose efficiently elicits functional antibodies.

**Figure 3:**
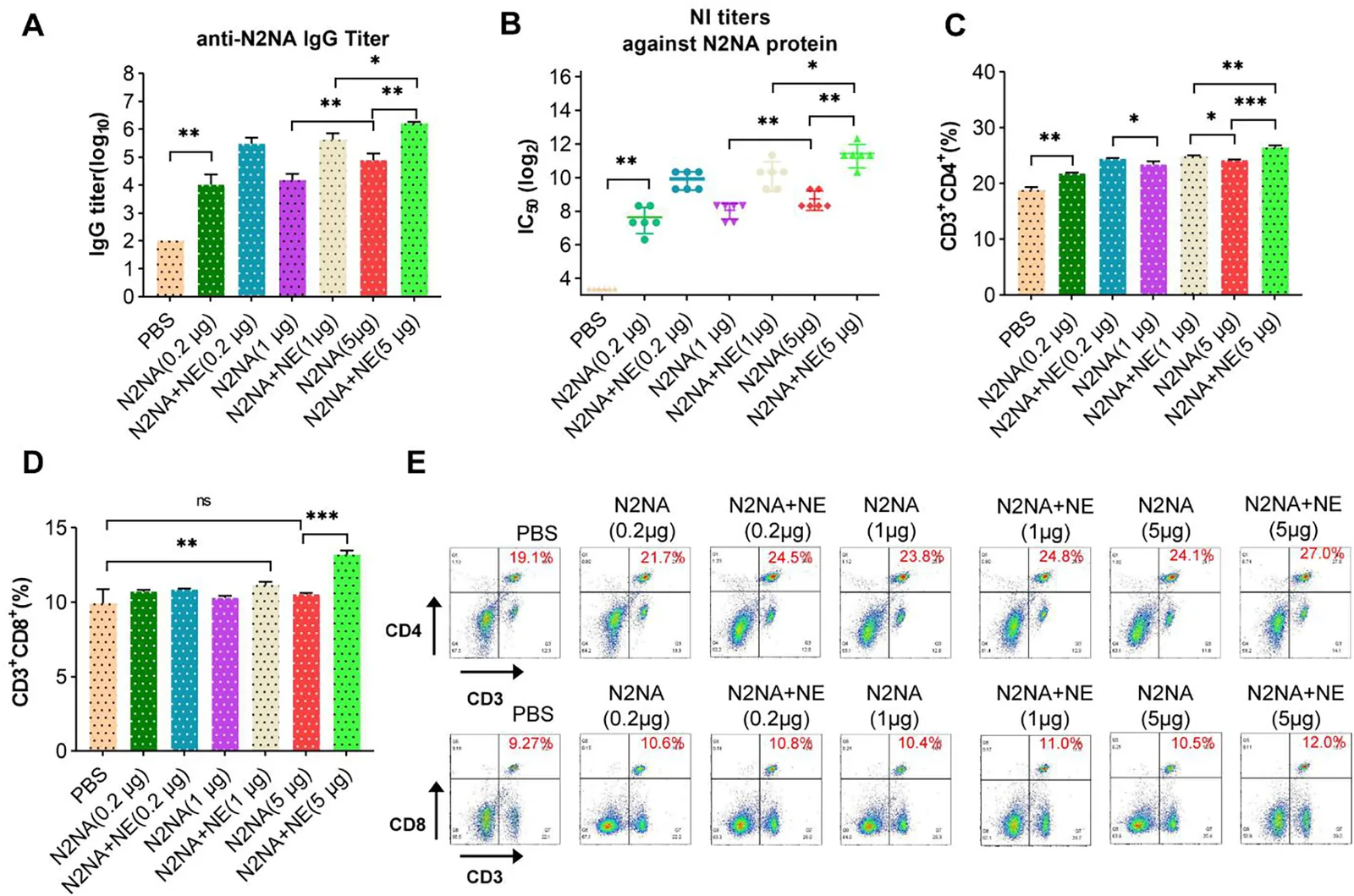
Humoral and cellular immune responses elicited by N2NA vaccine at different dosages with or without NE adjuvant. **(A)** ELISA analysis of antibody titers; **(B)** NI assay for functional antibody detection; **(C)** Proportions of CD3^+^CD4^+^ T cell subsets; **(D)** Proportions of CD3^+^CD8^+^ T cell subsets; **(E)** Representative flow cytometric plots of T lymphocyte subsets. * indicates P<0.05 (statistically significant). "**" indicates P<0.01 (statistically very significant), "***" indicates P < 0.001 (highly significant statistically), "ns" indicates not significant' (P ≥ 0.05).

Flow cytometric analysis of splenic T-cell subsets (**Figures 3C–E**) demonstrated that NE significantly enhanced both CD3^+^CD4^+^ and CD3^+^CD8^+^ T-cell responses, reaching the peak in the 5 μg N2NA+NE group. Activation of CD3^+^CD8^+^ T-cell exhibited clear adjuvant dependence: no significant difference was observed between non-adjuvanted groups and the PBS control, whereas the 1 μg N2NA+NE group exhibited a higher CD8^+^ T-cell proportion than the 5 μg non-adjuvanted N2NA group. These results indicate that NE effectively compensates for the insufficient cellular immunity induced by low-dose antigen, greatly improving the overall immunogenicity of N2NA vaccine.

### 3.4 Monovalent N1NA and BVNA elicit specific immune responses, and NE adjuvant significantly enhances viral challenge protection

Using recombinant proteins matched to the immunogens as detection antigens, ELISA and NI assays (**Figures 4A–D**) showed that NE adjuvant significantly elevated specific binding antibody and functional NI antibody titers induced by both N1NA and BVNA. Antibody levels in non-adjuvanted groups were markedly lower than those in adjuvanted groups, confirming the potent humoral immunity-enhancing effect of NE on both monovalent NA vaccines.

**Figure 4:**
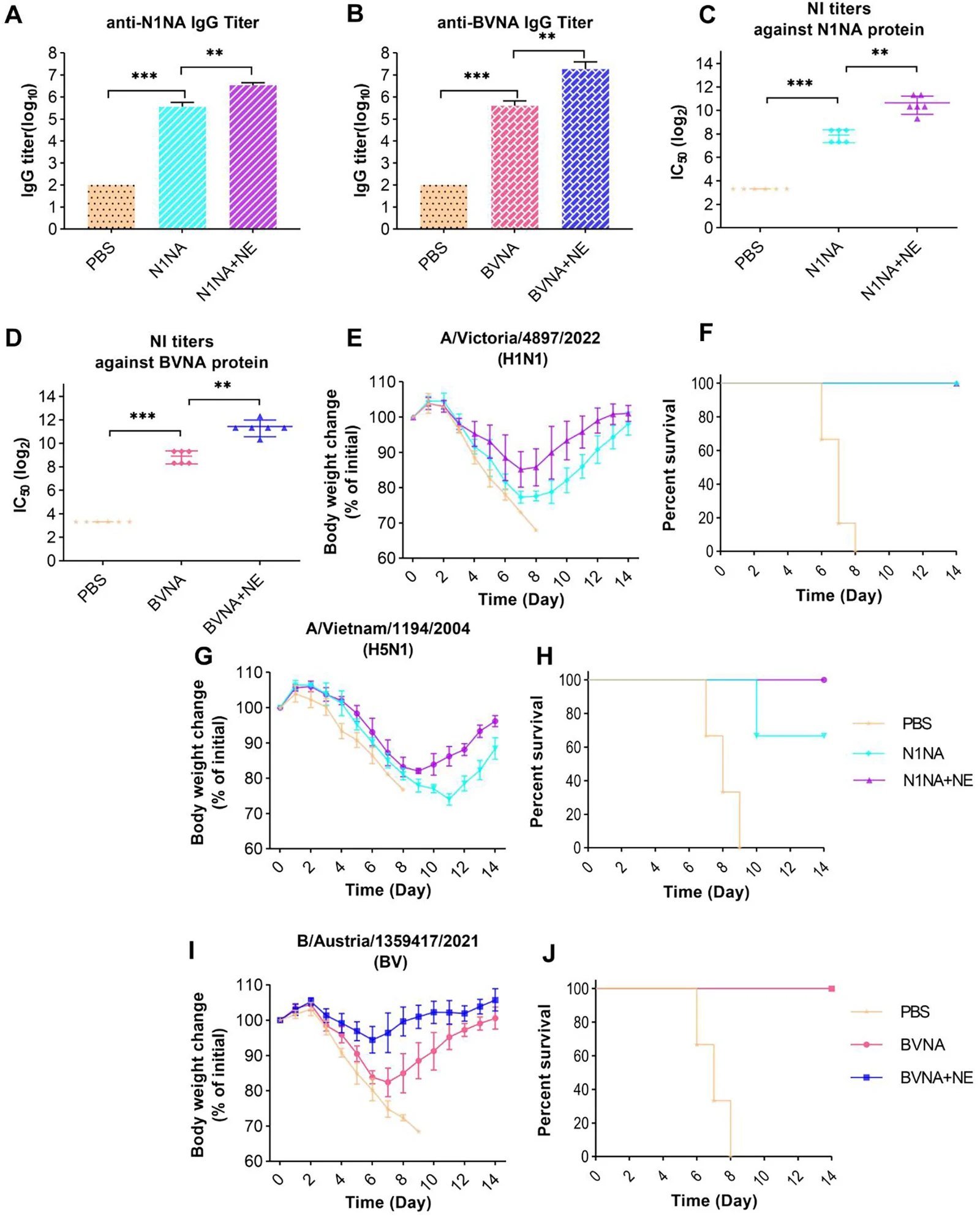
Humoral immune responses and protective efficacy of monovalent N1NA and BVNA vaccines with or without NE adjuvant. **(A)** ELISA analysis of antibody titers induced by N1NA vaccine; **(B)** ELISA analysis of antibody titers induced by BVNA vaccine; **(C)** NI assay for functional antibody detection of N1NA vaccine; **(D)** NI assay for functional antibody detection of BVNA vaccine; **(E)** Body weight changes of N1NA-immunized mice following homologous H1N1 challenge; **(F)** Survival rates of N1NA-immunized mice following homologous H1N1 challenge; **(G)** Body weight changes of N1NA-immunized mice following heterologous H5N1 challenge; **(H)** Survival rates of N1NA-immunized mice following heterologous H5N1 challenge; **(I)** Body weight changes of BVNA-immunized mice following homologous BV challenge; **(J)** Survival rates of BVNA-immunized mice following homologous BV challenge. ** indicates P<0.01 (statistically very significant), *** indicates P<0.001 (highly significant statistically).

Viral challenge experiments (**Figures 4E–J**) demonstrated that after homologous H1N1 challenge, mice in both N1NA and N1NA+NE groups exhibited transient clinical signs, including reduced activity, ruffled fur, and a hunched posture, together with body-weight loss. Both groups reached the lowest body weight on day 7 followed by gradual recovery, with 100% survival in both groups, while all PBS control mice died by day 8. Against heterologous H5N1 challenge, the N1NA+NE group showed an earlier weight nadir, faster recovery and no mortality, whereas 2 deaths occurred in the non-adjuvanted N1NA group. All BVNA-immunized mice survived homologous BV challenge, and the adjuvanted group exhibited milder weight loss and significantly faster recovery than the non-adjuvanted group. Overall, monovalent N1NA and BVNA vaccines provide effective antiviral protection, and NE adjuvant further reduces infection-related weight loss, accelerates recovery, and significantly improves cross-protection against heterologous H5N1 virus.

### 3.5 Trivalent NA vaccine induces balanced multi-subtype immunity, and NE adjuvant confers broad and potent cross-protection

To systematically evaluate the immunogenicity and protective capacity of trivalent NA vaccines formulated with or without NE adjuvant, we detected N1-, N2- and BV NA-specific binding antibodies as well as functional NI antibody titers. ELISA results (**Figures 5A–C**) demonstrated that both Tri-NA and Tri-NA+NE groups could efficiently induce specific binding antibodies targeting the three NA subtypes, and antibody levels were significantly higher in the adjuvanted group. Compared with the monovalent NA immunization results above, each subtype-specific antibody titer elicited by the trivalent vaccine was basically consistent with that induced by the corresponding monovalent vaccine alone, indicating no obvious immune competition inhibition among the three co-administered antigens. NI functional antibody assays showed fully consistent trends with ELISA results, and the Tri-NA+NE group possessed the optimal NI antibody titers (**Figures 5D–F**).

**Figure 5:**
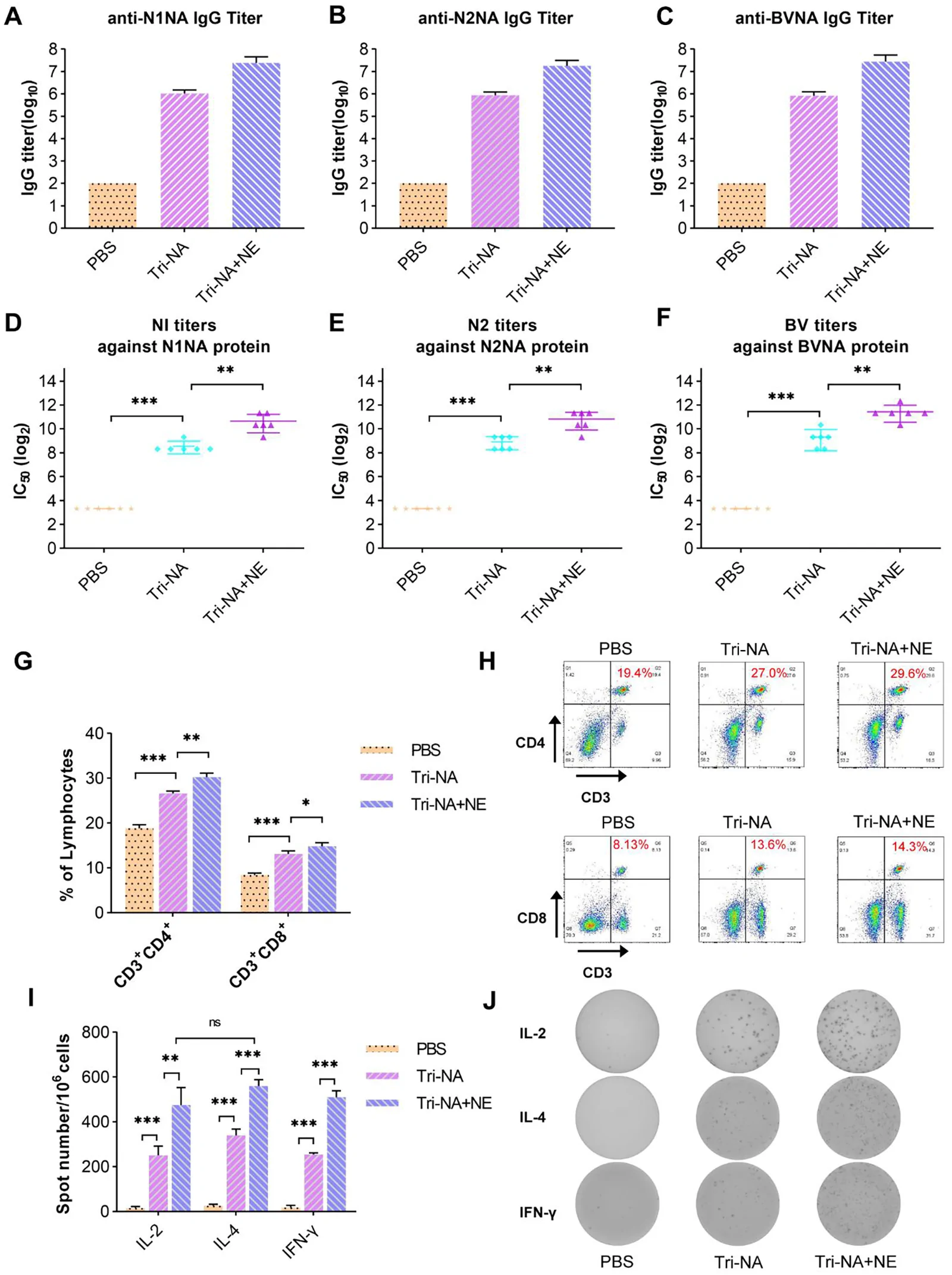
Humoral and cellular immune responses induced by trivalent NA vaccine with or without NE adjuvant. **(A)** ELISA analysis of N1 NA antibody titers; **(B)** ELISA analysis of N2 NA antibody titers; **(C)** ELISA analysis of BV NA antibody titers; **(D)** NI assay for N1 NA functional antibody detection; **(E)** NI assay for N2 NA functional antibody detection; **(F)** NI assay for BV NA functional antibody detection; **(G)** Proportions of CD3^+^CD4^+^ and CD3^+^CD8^+^ T cell subsets; **(H)** Representative flow cytometric plots of T lymphocyte subsets; **(I)** Quantification of cytokine-secreting splenocytes by ELISPOT assay; **(J)** Representative ELISPOT images of cytokine-secreting lymphocytes. * indicates P<0.05 (statistically significant). "**" indicates P<0.01 (statistically very significant), "***" indicates P < 0.001 (highly significant statistically), "ns" indicates not significant' (P ≥ 0.05).

Flow cytometric analysis (**Figures 5G, H**) indicated that NE adjuvant significantly boosted CD3^+^CD4^+^ and CD3^+^CD8^+^ T cell responses. ELISpot assays using mixed trivalent NA proteins as stimuli (**Figures 5I, J**) revealed that the Tri-NA+NE group had significantly more IL-2-, IL-4- and IFN-γ-secreting lymphocytes than the non-adjuvanted Tri-NA group. Moreover, there was no significant difference in the secretion levels of the three cytokines in the Tri-NA+NE group, which could simultaneously trigger balanced Th1 and Th2 cellular immune responses.

Viral challenge tests were carried out using five influenza strains of different lineages (**Figure 6**), including two H1N1 strains (A/Victoria/4897/2022, A/Guangdong-Maonan/SWL1536/2019), two B/Victoria lineage strains (B/Austria/1359417/2021, B/Washington/02/2019) and one highly pathogenic H5N1 strain (A/Vietnam/1194/2004). Combined analysis of body weight changes, clinical signs, and survival curves showed that the Tri-NA+NE group achieved 100% mouse survival against all five viral challenges, with milder clinical responses, slighter body weight loss and faster recovery after infection. The non-adjuvanted Tri-NA vaccine could provide effective protection against four seasonal influenza viruses, whereas mouse death was observed after heterologous H5N1 viral challenge. These results confirm that the trivalent NA vaccine alone can provide a moderate broad-spectrum protection, and that the NE adjuvant can further reduce infection-induced damage and enhance cross-protective efficacy against highly pathogenic heterologous influenza viruses.

**Figure 6:**
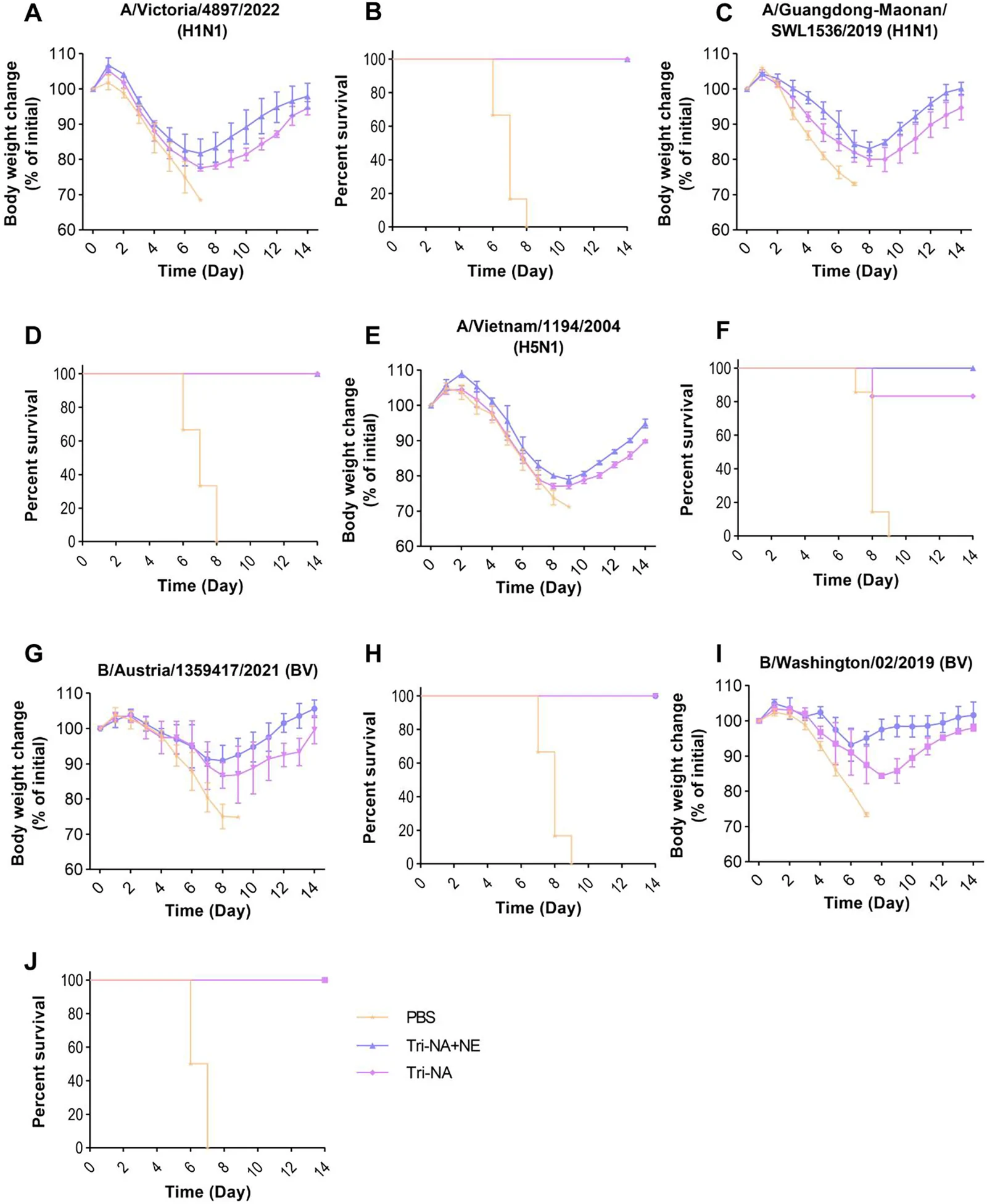
Protective efficacy of trivalent NA vaccine with or without NE adjuvant against five divergent influenza strains. **(A, C, E, G, I)** Body weight changes of immunized mice after viral challenge with H1N1 (A/Victoria/4897/2022), H1N1 (A/Guangdong-Maonan/SWL1536/2019), H5N1 (A/Vietnam/1194/2004), B/Victoria (B/Austria/1359417/2021) and B/Victoria (B/Washington/02/2019), respectively; **(B, D, F, H, J)** Corresponding survival curves of mice challenged with the above five influenza strains.

## 4 Discussion

HA and NA are the two major surface glycoproteins of influenza viruses, coordinating the viral infection cycle and serving as the primary antigens targeted by host immune responses. The two glycoproteins carry out distinct biological functions: HA mediates viral attachment and membrane fusion to drive host cell entry (Benton et al., 2020), whereas NA hydrolyzes cell-surface sialic acid residues to release progeny virions and facilitate viral spread through respiratory mucus (de Vries et al., 2020). All licensed seasonal influenza vaccines adopt a uniform quality control criterion of 15 μg HA per subtype (Fiore et al., 2010), yet no standardized benchmarks have been defined for NA content, structural integrity or enzymatic activity. Natural influenza infection elicits balanced anti-HA and anti-NA immunity; by contrast, commercial vaccines only induce weak NA-specific responses (Couch et al., 2012; Ito et al., 2020), owing to the inherent instability of NA, inconsistent NA antigen quantities across manufacturers, and conformational destruction triggered by formalin or EDTA inactivation treatments.

Mounting epidemiological and preclinical evidence indicates that NA-directed immunity confers autonomous anti-influenza protection and extends the breadth of immune defense when combined with HA-mediated responses (Ramos et al., 2024). Compared with HA, which is prone to frequent antigenic drift, NA exhibits a markedly slower evolutionary rate; its catalytic domain and framework residues remain highly conserved across influenza A and B subtypes (Sandbulte et al., 2011; Creytens et al., 2021). Epidemiological data from past pandemics provide human evidence of NA’s protective capacity: pre-existing N2-specific antibodies elicited by H2N2 reduced susceptibility to the 1968 H3N2 Hong Kong influenza strain (Monto and Kendal, 1973), whereas serum NI antibody titers independently correlated with attenuated symptoms and truncated viral shedding during the 2009 H1N1 pandemic (Couch et al., 2013), a beneficial phenotype not associated with HAI antibodies. Cumulative investigations of NA-targeted monoclonal antibodies further establish NA as an attractive antigen candidate for universal influenza vaccine development (Lei et al., 2023; Momont et al., 2023).

Full-length recombinant N1, N2 and B/Victoria NA matching 2024–2025 WHO vaccine strains were generated using a baculovirus-insect cell platform. Biochemical and electrophoretic analyses confirmed functional NA multimers, a key structure supporting potent immunogenicity. Murine experiments showed prime-boost vaccination induced stronger humoral and cellular immunity than single-dose immunization. NE adjuvant greatly boosted NA-specific IgG and NI antibodies; 1 μg adjuvanted NA outperformed 5 μg unadjuvanted antigen to overcome low-dose immunogenicity defects. The NE-adjuvanted prime-boost regimen expanded CD4^+^/CD8^+^ T cell pools and increased IL-2, IL-4 and IFN-γ secretion, generating balanced Th1/Th2 immunity for broad antiviral protection. Monovalent N1 and B/Victoria NA fully protected mice against homologous infection, while NE relieved weight loss, accelerated recovery and improved cross-protection against heterologous H5N1. Trivalent co-immunization with three NA subtypes produced equivalent subtype-specific antibodies without antigenic competition, extending Johansson and Kilbourne’s N1/N2 findings to influenza B NA (Johansson and Kilbourne, 1993). Viral challenge assays revealed unadjuvanted trivalent NA protected mice against four seasonal strains but caused death after H5N1 infection. Conversely, NE-adjuvanted trivalent NA secured 100% survival against all five tested strains with milder weight loss, proving NE expands cross-subtype protection of multivalent NA vaccines.

This multivalent NA vaccine exhibits promising protective potency, yet several limitations remain. First, only monovalent NA vaccines were assessed, with no HA-NA combinatorial formulations developed. Concurrent anti-HA and anti-NA immune responses confer superior protection, yet mixed antigens may trigger immunodominance competition that requires optimized HA/NA ratios (Krammer et al., 2018b). Lacking combinatorial experimental groups, this study cannot evaluate their synergistic protective effects. Second, all immunization and viral challenge assays were restricted to BALB/c mice. No validation was carried out in ferrets or guinea pigs, which better recapitulate human influenza respiratory infection, hindering the translational potential of this trivalent NA vaccine (Bodewes et al., 2010; Bouvier and Lowen, 2010). Third, only NI antibody activity and T-cell cytokine secretion were measured, while NA-mediated ADCC and CDC effector pathways were uncharacterized, resulting in incomplete elucidation of NA-driven immune protective mechanisms (Sylte and Suarez, 2009). Additionally, thermal and storage stability of NA antigens and vaccine formulations were also not assessed in this study, requiring further investigation in future work.

To tackle the aforementioned drawbacks, we plan a set of follow-up laboratory investigations centered on mechanistic exploration and expanded preclinical evaluation. We first aim to comprehensively characterize NA-triggered ADCC and CDC effector functions, which will clarify the multifaceted protective effects of recombinant NA antibodies beyond NI-mediated inhibition. Inspired by the combinatorial immunization framework reported by Johansson et al (Johansson, 1999), future trials will incorporate our recombinant NA antigens alongside HA to screen balanced antigen ratios that mitigate immunodominance interference without compromising HAI antibody production. Additionally, further *in vivo* testing in ferret and guinea pig models is scheduled to assess the protective breadth and persistence of the NE-adjuvanted trivalent NA vaccine in animal models that more closely mirror human influenza infection physiology.

## 5 Conclusion

This study established a baculovirus-based platform for generating multi-subtype recombinant NA antigens. We validated that NE adjuvant strengthens cross-subtype protection of multivalent NA vaccines, offering a viable strategy for universal influenza vaccine research. Co-immunization with different NA subtypes induced no antigenic competition, which provides experimental support for incorporating standardized NA moieties into influenza vaccine designs to broaden protective coverage.

## Data Availability

The original contributions presented in the study are included in the article/supplementary material. Further inquiries can be directed to the corresponding authors.

## References

[B15] Benton D. J. Gamblin S. J. Rosenthal P. B. Skehel J. J. (2020). Structural transitions in influenza haemagglutinin at membrane fusion pH. Nature 583, 150–153. doi: 10.1038/s41586-020-2333-6 32461688 PMC7116728

[B29] Bodewes R. Rimmelzwaan G. F. Osterhaus A. D. (2010). Animal models for the preclinical evaluation of candidate influenza vaccines. Expert Rev. Vaccines 9, 59–72. doi: 10.1586/erv.09.148 20021306

[B30] Bouvier N. M. Lowen A. C. (2010). Animal models for influenza virus pathogenesis and transmission. Viruses 2, 1530–1563. doi: 10.3390/v20801530 21442033 PMC3063653

[B1] Cargnin Faccin F. Perez D. R. (2024). Pandemic preparedness through vaccine development for avian influenza viruses. Hum. Vaccin Immunother. 20, 2347019. doi: 10.1080/21645515.2024.2347019 38807261 PMC11141480

[B8] Chen Y. Q. Wohlbold T. J. Zheng N. Y. Huang M. Huang Y. Neu K. E. . (2018). Influenza infection in humans induces broadly cross-reactive and protective neuraminidase-reactive antibodies. Cell 173, 417–429.e410. doi: 10.1016/j.cell.2018.03.030 29625056 PMC5890936

[B12] Coffman R. L. Sher A. Seder R. A. (2010). Vaccine adjuvants: putting innate immunity to work. Immunity 33, 492–503. doi: 10.1016/j.immuni.2010.10.002 21029960 PMC3420356

[B24] Couch R. B. Atmar R. L. Franco L. M. Quarles J. M. Wells J. Arden N. . (2013). Antibody correlates and predictors of immunity to naturally occurring influenza in humans and the importance of antibody to the neuraminidase. J. Infect. Dis. 207, 974–981. doi: 10.1093/infdis/jis935 23307936 PMC3633450

[B18] Couch R. B. Atmar R. L. Keitel W. A. Quarles J. M. Wells J. Arden N. . (2012). Randomized comparative study of the serum antihemagglutinin and antineuraminidase antibody responses to six licensed trivalent influenza vaccines. Vaccine 31, 190–195. doi: 10.1016/j.vaccine.2012.10.065 23107591 PMC3520601

[B22] Creytens S. Pascha M. N. Ballegeer M. Saelens X. de Haan C. A. M. (2021). Influenza neuraminidase characteristics and potential as a vaccine target. Front. Immunol. 12, 786617. doi: 10.3389/fimmu.2021.786617 34868073 PMC8635103

[B16] de Vries E. Du W. Guo H. de Haan C. A. M. (2020). Influenza A virus hemagglutinin-neuraminidase-receptor balance: preserving virus motility. Trends Microbiol. 28, 57–67. doi: 10.1016/j.tim.2019.08.010 31629602 PMC7172302

[B5] Eichelberger M. C. Morens D. M. Taubenberger J. K. (2018). Neuraminidase as an influenza vaccine antigen: a low hanging fruit, ready for picking to improve vaccine effectiveness. Curr. Opin. Immunol. 53, 38–44. doi: 10.1016/j.coi.2018.03.025 29674167 PMC6141346

[B17] Fiore A. E. Uyeki T. M. Broder K. Finelli L. Euler G. L. Singleton J. A. . (2010). Prevention and control of influenza with vaccines: recommendations of the Advisory Committee on Immunization Practices (ACIP), 2010. MMWR Recomm Rep. 59, 1–62. Available online at: https://pubmed.ncbi.nlm.nih.gov/20689501/. 20689501

[B2] Gamblin S. J. Skehel J. J. (2010). Influenza hemagglutinin and neuraminidase membrane glycoproteins. J. Biol. Chem. 285, 28403–28409. doi: 10.1074/jbc.R110.129809 20538598 PMC2937864

[B9] Hansen L. McMahon M. Turner H. L. Zhu X. Turner J. S. Ozorowski G. . (2023). Human anti-N1 monoclonal antibodies elicited by pandemic H1N1 virus infection broadly inhibit HxN1 viruses *in vitro* and *in vivo*. Immunity 56, 1927–1938.e1928. doi: 10.1016/j.immuni.2023.07.004 37506693 PMC10529248

[B19] Ito H. Nishimura H. Kisu T. Hagiwara H. Watanabe O. Kadji F. M. N. . (2020). Low response in eliciting neuraminidase inhibition activity of sera among recipients of a split, monovalent pandemic influenza vaccine during the 2009 pandemic. PloS One 15, e0233001. doi: 10.1371/journal.pone.0233001 32401814 PMC7219752

[B32] Johansson B. E. (1999). Immunization with influenza A virus hemagglutinin and neuraminidase produced in recombinant baculovirus results in a balanced and broadened immune response superior to conventional vaccine. Vaccine 17, 2073–2080. doi: 10.1016/s0264-410x(98)00413-7 10217609

[B27] Johansson B. E. Kilbourne E. D. (1993). Dissociation of influenza virus hemagglutinin and neuraminidase eliminates their intravirionic antigenic competition. J. Virol. 67, 5721–5723. doi: 10.1128/jvi.67.10.5721-5723.1993 8371337 PMC237988

[B28] Krammer F. Fouchier R. A. M. Eichelberger M. C. Webby R. J. Shaw-Saliba K. Wan H. . (2018b). NAction! How can neuraminidase-based immunity contribute to better influenza virus vaccines? mBio 9, e02332-17. doi: 10.1128/mBio.02332-17 29615508 PMC5885027

[B3] Krammer F. Smith G. J. D. Fouchier R. A. M. Peiris M. Kedzierska K. Doherty P. C. . (2018a). Influenza. Nat. Rev. Dis. Primers 4, 3. doi: 10.1038/s41572-018-0002-y 29955068 PMC7097467

[B25] Lei R. Kim W. Lv H. Mou Z. Scherm M. J. Schmitz A. J. . (2023). Leveraging vaccination-induced protective antibodies to define conserved epitopes on influenza N2 neuraminidase. Immunity 56, 2621–2634.e2626. doi: 10.1016/j.immuni.2023.10.005 37967533 PMC10655865

[B13] Loganathan M. Francis B. Krammer F. (2024). Production of influenza virus glycoproteins using insect cells. Methods Mol. Biol. 2762, 43–70. doi: 10.1007/978-1-0716-3666-4_4 38315359

[B7] Lv K. Li X. Zhu X. Cai J. P. Liu S. Sun Y. . (2025). Broad neuraminidase antibodies confer protection against seasonal and avian influenza viruses. Nat. Commun. 16, 7103. doi: 10.1038/s41467-025-62040-1 40753167 PMC12318061

[B11] Memoli M. J. Shaw P. A. Han A. Czajkowski L. Reed S. Athota R. . (2016). Evaluation of antihemagglutinin and antineuraminidase antibodies as correlates of protection in an influenza A/H1N1 virus healthy human challenge model. mBio 7, e00417-00416. doi: 10.1128/mBio.00417-16 27094330 PMC4959521

[B26] Momont C. Dang H. V. Zatta F. Hauser K. Wang C. di Iulio J. . (2023). A pan-influenza antibody inhibiting neuraminidase via receptor mimicry. Nature 618, 590–597. doi: 10.1038/s41586-023-06136-y 37258672 PMC10266979

[B23] Monto A. S. Kendal A. P. (1973). Effect of neuraminidase antibody on Hong Kong influenza. Lancet 1, 623–625. doi: 10.1016/s0140-6736(73)92196-x 4121842

[B20] Ramos K. E. Okba N. M. A. Tan J. Bandawane P. Meade P. S. Loganathan M. . (2024). Broadly protective bispecific antibodies that simultaneously target influenza virus hemagglutinin and neuraminidase. mBio 15, e0108524. doi: 10.1128/mbio.01085-24 38899870 PMC11253627

[B10] Sandbulte M. R. Jimenez G. S. Boon A. C. Smith L. R. Treanor J. J. Webby R. J. (2007). Cross-reactive neuraminidase antibodies afford partial protection against H5N1 in mice and are present in unexposed humans. PloS Med. 4, e59. doi: 10.1371/journal.pmed.0040059 17298168 PMC1796909

[B21] Sandbulte M. R. Westgeest K. B. Gao J. Xu X. Klimov A. I. Russell C. A. . (2011). Discordant antigenic drift of neuraminidase and hemagglutinin in H1N1 and H3N2 influenza viruses. Proc. Natl. Acad. Sci. U.S.A. 108, 20748–20753. doi: 10.1073/pnas.1113801108 22143798 PMC3251064

[B14] Strohmeier S. Amanat F. Campbell J. D. Traquina P. Coffman R. L. Krammer F. (2022b). A CpG 1018 adjuvanted neuraminidase vaccine provides robust protection from influenza virus challenge in mice. NPJ Vaccines 7, 81. doi: 10.1038/s41541-022-00486-w 35869085 PMC9305062

[B6] Strohmeier S. Amanat F. Carreño J. M. Krammer F. (2022a). Monoclonal antibodies targeting the influenza virus N6 neuraminidase. Front. Immunol. 13, 944907. doi: 10.3389/fimmu.2022.944907 35967389 PMC9363587

[B31] Sylte M. J. Suarez D. L. (2009). Influenza neuraminidase as a vaccine antigen. Curr. Top. Microbiol. Immunol. 333, 227–241. doi: 10.1007/978-3-540-92165-3_12 19768409

[B4] Wohlbold T. J. Krammer F. (2014). In the shadow of hemagglutinin: a growing interest in influenza viral neuraminidase and its role as a vaccine antigen. Viruses 6, 2465–2494. doi: 10.3390/v6062465 24960271 PMC4074938

